# Radiation and Immune Checkpoint Inhibitors: Combination Therapy for Treatment of Hepatocellular Carcinoma

**DOI:** 10.3390/ijms242316773

**Published:** 2023-11-26

**Authors:** Perla Chami, Youssef Diab, Danny N. Khalil, Hassan Azhari, William R. Jarnagin, Ghassan K. Abou-Alfa, James J. Harding, Joseph Hajj, Jennifer Ma, Maria El Homsi, Marsha Reyngold, Christopher Crane, Carla Hajj

**Affiliations:** 1 Faculty of Medicine, American University of Beirut, Beirut 1107, Lebanon; pnc00@mail.aub.edu; 2 Faculty of Medicine, University of Balamand, Beirut 1100, Lebanon; youssef.diab@std.balamand.edu.lb (Y.D.);; 3 Memorial Sloan Kettering Cancer Center, New York, NY 10027, USA; 4 Department of Surgery, Weill Medical College, Cornell University, New York, NY 10021, USA; 5 Department of Medicine, Weill Medical College, Cornell University, New York, NY 10021, USA; 6 New York Proton Center, New York, NY 10035, USA

**Keywords:** hepatocellular carcinoma, immunotherapy, immune checkpoint inhibitors, radiation therapy, combination therapy

## Abstract

The liver tumor immune microenvironment has been thought to possess a critical role in the development and progression of hepatocellular carcinoma (HCC). Despite the approval of immune checkpoint inhibitors (ICIs), such as programmed cell death receptor 1 (PD-1)/programmed cell death ligand 1 (PD-L1) and cytotoxic T lymphocyte associated protein 4 (CTLA-4) inhibitors, for several types of cancers, including HCC, liver metastases have shown evidence of resistance or poor response to immunotherapies. Radiation therapy (RT) has displayed evidence of immunosuppressive effects through the upregulation of immune checkpoint molecules post-treatment. However, it was revealed that the limitations of ICIs can be overcome through the use of RT, as it can reshape the liver immune microenvironment. Moreover, ICIs are able to overcome the RT-induced inhibitory signals, effectively restoring anti-tumor activity. Owing to the synergetic effect believed to arise from the combination of ICIs with RT, several clinical trials are currently ongoing to assess the efficacy and safety of this treatment for patients with HCC.

## 1. Introduction

Hepatocellular carcinoma (HCC) is a prominent health issue with an increasing rate of worldwide occurrence [1,2,3]. In 2020, HCC accounted for 906,000 new cases and 830,000 new deaths, making it the third greatest cause of cancer death [1]. Liver diseases, such as cirrhosis and chronic viral hepatitis, often result in the development of inflammation, fibrosis, necrosis, and subsequent regeneration, which will injure and alter the organization of the liver tissues, contributing to HCC prognosis [4,5]. The main risk factors include hepatitis C virus (HCV), hepatitis B virus (HBV), alcohol use disorder, and non-alcoholic steatohepatitis (NASH) [6]. Locoregional therapies such as ablation, arterially directed therapies, and external beam radiation treatment are often indicated for cases of early- or intermediate-stage HCC [7,8,9]. Systemic therapies are used for more advanced stages [10] and include multityrosine kinase inhibitors (mTKIs) and immune checkpoint inhibitors (ICIs) [7]. Recent advancements in clinical trials and approvals by the United States Food and Drug Administration (FDA) led to a shift from relying on the multikinase inhibitor sorafenib as the frontline treatment for HCC treatment in favor of ICIs, either as single agents or in combinations such as atezolizumab and bevacizumab [11] or durvalumab plus tremelimumab [12].

The immune microenvironment of the liver is modified by HCC [13]. It deregulates the immune system by altering cytokines, immune cells, inhibitory receptors, and their ligands [14], which in turn promotes tumor survival and proliferation [15]. Some of the ways in which radiotherapy advances anti-tumor activity are by promoting T cell immunity, releasing tumor antigens, and generating reactive oxygen species (ROS) that augment anti-tumor immunity [13,16,17].

Radiation therapy (RT) may also cause some immunosuppressive effects, such as the upregulation of immune checkpoint molecules on cytotoxic T cells or on the tumor cells [18]. The addition of ICIs to radiation can help overcome these effects, bringing about a synergistic outcome. RT also causes the regression of cancer cells outside of the field of radiation, a phenomenon referred to as an abscopal effect. It has been suggested that ICIs can intensify this effect [19]. Currently, there is no consensus on the ideal timing sequence for the combination of ICIs and RT. Understanding the hepatic immune microenvironment and the mechanism of action of ICIs and RT may provide some insight into the optimal combination sequence.

A literature search was conducted through PubMed and Google Scholar to look for the studies relating to the combination therapy of RT plus ICIs in the treatment of HCC. The keywords included were the names of each ICI that has been used to treat HCC along with the words: “radiation”, “combination”, “immunotherapy”, and “hepatocellular carcinoma”.

In this review, we will discuss what makes the liver an immunogenic organ and the implications on possible treatment plans. Secondly, we will explore the available ICIs for HCC treatment, as well as the differences between monotherapy and combination therapy. Thirdly, we will go into the different types of radiation therapies available and the immune system’s response to them. Finally, we will investigate the potentially synergetic effect of combining RT and ICIs by examining relevant clinical trials.

## 2. The Hepatic Immune Microenvironment

The liver is an organ that has the ability to mount an immune response as it is rich in immune cells; thus, it creates a link between the gastrointestinal (GI) and immune systems, allowing the liver to respond to pathogens incoming from the GI tract [20]. The small diameters of the sinusoidal structures of liver cells along with small rises in systemic venous pressure result in the stasis of blood flow, which favors the presentation of antigens to immune cells and facilitates extravasation of lymphocytes [21]. The immune cells resident in the liver include natural killer cells, natural killer T cells and Kupffer cells (macrophages) belonging to the innate immune response, and B and T cells (e.g., CD4^+^ and CD8^+^ T cells) belonging to the adaptive immune response [21].

In case of lesions to the liver, Kupffer cells promote cytokine and chemokine production, which triggers the recruitment of immune cells [22]. More specifically, evasion of the immune system and tumor development by Kupffer cells and other monocyte-derived tumor-associated macrophages can occur through different routes, such as the release of immunosuppressive cytokines (e.g., interleukin-10 (IL-10), transforming growth factor-β (TGF-β)), the expression of programmed cell death ligand 1 (PD-L1), the downregulation of class II major histocompatibility complex (MHC-II) and costimulatory markers (e.g., CD80, CD86), the recruitment of CD4^+^ T helper cells expressing IL-17 and regulatory T cells, and the triggering of angiogenesis. In patients with HCC, the overexpression of immune checkpoint markers, such as PD-L1, programmed cell death receptor 1 (PD-1), and cytotoxic T lymphocyte associated protein 4 (CTLA-4), on cells results in T cell exhaustion [23]. Dendritic cells, as antigen-presenting cells (APCs), possess the ability to intercept antigens in circulation and traffic them to T cells in draining lymph nodes [21]. In HCC patients, this process is hindered as tumor cells release cytokines and chemokines that recruit high levels of CD4^+^CD25^+^FoxP3^+^ regulatory T cells, which are accompanied by a decrease in CD8^+^ T cell infiltration at the tumor sites and a significant reduction in CD8^+^ T cell expression of granzyme A, granzyme B, and perforin. These events were found to correlate with compromised immunity, HCC progression, and increased mortality [24]. Similarly, a study looking to characterize myeloid-derived suppressor cells (MDSCs) in patients with HCC found that they were present at significantly higher levels in the tumors and peripheral blood of HCC patients compared to healthy individuals and that upon coculture with autologous CD4^+^ T cells they promoted the expansion of CD4^+^CD25^+^FoxP3^+^ regulatory T cells and inhibited T cell proliferation and activation [25]. In addition, patients with breast, prostate, and colorectal cancers, melanomas, or non-small cell lung cancers with liver metastases were found to have a reduction in the number of intratumoral T cell clones, a decreased diversity of T cells, and a reduced effector function of CD8^+^ T cells. These findings were not observed in cancers with lung metastases [13].

In murine models, liver metastases were also shown to siphon active CD8^+^ T cells away from systemic circulation, to reduce the number of CD4^+^ T cells, and to recruit immunosuppressive macrophages that induce apoptosis of antigen-specific T cells in the liver. These high levels of hepatic CD11b^+^F4/80^+^ myeloid cells in liver metastases are suggested to be involved in resistance to treatment with immunotherapy as the activation of the T cell apoptosis pathway was revealed to occur by direct cell contact with myeloid cells through the binding of the ligands Fas and FasL, which were found to be highly expressed in T cells and hepatic myeloid cells, respectively. Additionally, expression of FasL on hepatic macrophages was significantly higher than that of lung macrophages [13].

## 3. Immunotherapies for Hepatocellular Carcinoma

### 3.1. Immunotherapies Available for HCC

Following the approval of atezolizumab (anti-PD-L1) plus bevacizumab, an anti-vascular endothelial growth factor A (anti-VEGF-A), as first-line therapy for unresectable HCC in 2020, treatment with ICIs has generated growing interest for the treatment of HCC. The combination of atezolizumab plus bevacizumab in IMbrave150, a global, multicenter, open-label, phase 3 randomized trial, resulted in superior outcomes in terms of overall survival (OS) and progression-free survival (PFS) over the tyrosine kinase inhibitor sorafenib, which had previously been the standard of systemic therapy for patients with advanced HCC (OS at 12 months: 67.2% vs. 54.6%; median PFS: 6.8 months vs. 4.3 months; *p* < 0.001) [11]. Another phase 2/3 randomized trial investigating the combination of sintilimab (anti-PD-1) plus IBI305 (a bevacizumab biosimilar) as first-line therapy revealed increased OS and PFS over sorafenib (median OS: NA vs. 10.4 months; median PFS: 4.6 months vs. 2.8 months; *p* < 0.0001) in patients with unresectable HBV-related HCC [26]. In the more recent randomized, open-label, sponsor-blind, multicenter, global, phase 3 HIMALAYA trial, durvalumab (anti-PD-L1) monotherapy was found to be non-inferior to sorafenib (16.56 months vs. 13.77 months, respectively; *p* = 0.0035) and the combination of durvalumab plus tremelimumab (anti-CTLA-4) was shown to improve median OS over sorafenib (16.43 months vs. 13.77 months, respectively; *p* = 0.0035) in patients with unresectable HCC [12]. Updated findings were recently reported by the European Society for Medical Oncology (ESMO), in which the combination of durvalumab plus tremelimumab was found to yield a higher 4-year OS rate compared to sorafenib (25.2% vs. 15.1%, respectively) [27]. Currently, nivolumab (anti-PD-1) monotherapy, nivolumab plus ipilimumab (anti-CTLA-4), pembrolizumab (anti-PD-1), ramucirumab (anti-VEGF-R2), cabozantinib (mTKI), and regorafenib (mTKI) monotherapy are approved by the FDA as second-line therapy for unresectable HCC following the results of several clinical trials [28,29,30,31,32,33]. Results from a phase II study evaluating treatment with tislelizumab (anti-PD-1) as second-line therapy in patients with unresectable HCC have demonstrated an acceptable safety and tolerability profile with an overall response rate (ORR) of 12.4%, a median PFS of 2.7 months, and median OS of 12.4 months [34]. Tislelizumab is currently under study in comparison with sorafenib as first-line therapy in a phase 3 trial in patients with unresectable HCC (NCT03412773). Similarly, camrelizumab (anti-PD-1) was investigated as second-line treatment in a phase 2 trial for patients with advanced HCC and demonstrated anti-tumor activity with an ORR of 14.7%, median PFS of 2.1 months, and a median OS of 13.8 months [35]. A phase 3 trial looking to compare camrelizumab plus apatinib (a VEGF-R2 inhibitor) versus sorafenib as first-line therapy for patients with advanced HCC reported significantly improved outcomes in the combination group over sorafenib (median OS: 22.1 vs. 15.2 months; median PFS: 5.6 vs. 3.7 months, respectively; *p* < 0.0001) [36]. Moreover, toripalimab (anti-PD-1) plus anlotinib (a VEGF-R2 inhibitor) showed promising results as first-line therapy for patients with unresectable HCC with an ORR of 29%, median PFS of 11 months, and median OS of 18.2 months [37].

### 3.2. Mechanism of Action of Immune Checkpoint Inhibitors

Immune checkpoint molecules correspond to ligand–receptor pairs expressed on immune cells, APCs, and tumor cells possessing inhibitory or stimulatory functions, which serve to mediate the innate and adaptive immune responses. The presence of these molecules on tumor cells does not only facilitate escape from the immune system but is also involved in maintaining tumor activities, such as epithelial–mesenchymal transition, self-renewal, metastasis, resistance to anti-tumor drugs, angiogenesis, and anti-apoptosis, among others [38]. Examples of inhibitory immune checkpoint receptors include CTLA-4, PD-1, T cell immunoglobulin and mucin domain 3 (TIM-3), lymphocyte activation gene 3 (LAG-3), and killer immunoglobulin-like receptors (KIRs) [39].

The activation or inhibition of the immune response begins with the binding of the MHC receptors on T cells to the tumor-associated antigens (TAMs) on APCs. This process also requires additional costimulatory or coinhibitory binding associations, which constitute the basis of the immune checkpoint. For instance, the costimulatory marker CD28 on T cells binds to CD80 (B7-1) and CD86 (B7-2) on APCs. The result of both signals is the activation of the immune response as T cells proliferate and cytokines are released [40]. CTLA-4, which is expressed on active CD4^+^, CD8^+^, and regulatory T cells, is upregulated following T cell activation and acts as a competitive inhibitor of CD28 and binds to its receptors with higher affinity, thus diminishing T cell costimulation and curtailing the T cell response [40,41,42]. While its role under healthy conditions is to modulate T cell activity by inhibiting excessive T cell activation, CTLA-4, in the context of cancer, prevents the proliferation and activation of tumor-specific T cells [43]. Anti-CTLA-4 ICIs, such as ipilimumab and tremelimumab, bind to the CTLA-4 receptor in order to lift the inhibitory signal and favor the activation and proliferation of T cells and, therefore, the enhancement the immune response [40,44]. Similarly, anti-PD-1 and anti-PD-L1 ICIs serve to block binding at the PD-1/PD-L1 or PD-1/PD-L2 checkpoints and prevent the interaction between T cells and other immune cells (e.g., B cells, myeloid cells) expressing PD-1 and tumor cells, APCs, and other immune cells expressing PD-1 ligands [45]. In the absence of these ICIs, the binding between PD-1 and PD-L1 allows tumor cells to evade immune detection by the inactivation of T cells through the dephosphorylation of T cell-activating kinases [46]. In addition, the prevention of interleukin-2 (IL-2), interferon-𝛾 (IFN-𝛾), and tumor necrosis factor-𝛼 (TNF-𝛼) release and the decrease in T cell survival are characteristic of both CTLA-4 and PD-1 signaling. However, these pathways differ in the timing and location of their inhibitory functions as CTLA-4 acts in the lymph nodes early in the immune response during T cell priming while PD-1 generally acts later in the immune response during the T cell effector phase in peripheral tissues [45].

Table 1 summarizes the timing, location, and mechanisms of action of several ICIs that have been administered to patients with HCC.

### 3.3. Combination of Immune Checkpoint Inhibitors

Combinations of ICIs, such as atezolizumab plus bevacizumab and tremelimumab plus durvalumab, have shown promising additive anti-tumor activity for patients with HCC [11,12]. In the HIMALAYA trial, a single dose of tremelimumab (anti-CTLA-4) was given as a priming dose and was followed by durvalumab (anti-PD-L1), which was given every 4 weeks to patients with unresectable HCC [12]. Similarly, the CheckMate 040 trial aimed to assess different dosing plans of nivolumab (anti-PD-1) plus ipilimumab (anti-CTLA-4) in HCC patients that had received sorafenib treatment and reported the highest ORR in the treatment arm that received nivolumab plus a higher dose of ipilimumab (4 doses/every 3 weeks) followed by nivolumab every 2 weeks [29]. The rationale behind administering a priming dose of an CTLA-4 inhibitor followed by a PD-L1 inhibitor is that anti-CTLA-4 drugs can restore cytotoxic T cell activation in lymphoid tissues, which would consequently result in increased CD8^+^ T cell infiltration at the tumor site. In the absence of CTLA-4 blockers, the inhibition of the PD-1/PD-L1 checkpoint would not lead to anti-tumor activity as T cells would have been inactivated in the priming phase, and since they have not yet reached the effector phase they would be absent from tumor tissues [43]. In addition, the combination of VEGF blockers (e.g., bevacizumab or ramucirumab) with PD-1/PD-L1 ICIs has displayed the ability to remodel the immunosuppressive microenvironment characteristic of tumor growth. In the case of solid tumors, such as HCC, VEGF is secreted by hypoxic tumor cells and vascular endothelial cells, and leads to the recruitment of regulatory T cells, TAMs, and MDSCs, which in turn release additional VEGF and immunosuppressive cytokines (e.g., IL-10, TGF-β). VEGF also affects both the priming and effector stages by suppressing the maturation of dendritic cells and the presentation of antigens and by inhibiting the infiltration of activated T cells into cancer tissues, respectively. Consequently, anti-VEGF drugs facilitate the restoration of an immunostimulatory microenvironment. This is carried out through (1) a decrease in the production and release of regulatory T cells, TAMs, MDSCs, IL-10, and TGF-β, (2) the enhancement of CD8^+^ T cell activation in the priming stage by promoting dendritic cell antigen presentation, and (3) the normalization of tumor vasculature to allow the migration of activated T cells to the cancer. The addition of PD-1/PD-L1 ICIs serves to promote the anti-tumor response by activated T cells [47]. Currently, the TRIPLET-HCC phase 2/3 trial (NCT05665348) is planned to assess the efficacy and safety of combining an anti-CTLA-4 with an anti-PD-L1 and an anti-VEGF agent in patients with HCC by comparing treatment with ipilimumab plus atezolizumab and bevacizumab to the atezolizumab plus bevacizumab combination [48].

## 4. Radiotherapy

### 4.1. Immunological Effect of Radiation Therapy

#### 4.1.1. Effect on the Immune System

The tumor microenvironment (TME) of liver carcinoma comprises tumor cells, tumor-associated fibroblasts, hepatic non-parenchymal resident cells, and immune cells [49]. Tumor growth is facilitated by cytokines and chemokines that are secreted by the liver, as they create an immunosuppressive microenvironment by promoting angiogenesis, immune evasion, and a decreased incidence of apoptosis [50]. Radiotherapy can make the TME more susceptible to treatment by inducing immunogenic effects and several types of cell death such as necrosis, apoptosis, necroptosis, and autophagy [49].

Cell death causes DNA to accumulate in the cytosol which stimulates the production of type 1 interferons [51,52]. Type 1 interferons have been shown to confer anti-tumor activity by stimulating innate and adaptive immunity [53]. Radiation also promotes the release of danger-associated molecular patterns (DAMPs) and tumor antigens, activates APCs, and primes the T cells in lymph nodes [17]. Another important impact of RT is the production of ROS, which can alter macromolecules including proteins and DNA to increase their antigenicity [16]. Lastly, RT increases the immunogenicity of the tumor cells by increasing the expression of major histocompatibility complex class I (MHC-I) and FAS [54,55].

The term “abscopal effect” refers to the regression of tumors at distant non-irradiated sites [56]. It has been proposed that when radiation-induced immunogenic cell death occurs, DAMPs are released, which facilitate tumor antigen presentation [57]. Furthermore, there is an upregulation in antigen presentation due to the increase in MHC-I on tumor cells. This increase in antigen presentation is likely responsible for the abscopal effect [56].

#### 4.1.2. Radiation-Induced Liver Disease

Along with the killing of tumor cells, radiation causes tissue atrophy outside the tumor target site [56]. Radiation-induced liver disease (RILD) is a major drawback to the use radiation. It can occur at any time post-radiation, usually within the first 3 months after RT. Radiosensitive cells in the liver such as hepatic non-parenchymal cells, Kupffer cells, hepatic stellate cells, and sinusoidal endothelium cells secrete substances when they are radio-induced, which can result in liver fibrosis [58,59].

There are two types of RILD: classical and non-classical. Patients suffering from classical RILD exhibit fatigue, weight gain, abdominal pain, enlargement of the abdomen, anicteric ascites, and an increase in alkaline phosphatase levels. These symptoms usually appear 1–4 months post-RT [59,60,61]. An important marker of classical RILD is the elimination of the central vein lumina due to the trapping of erythrocytes in a matrix of reticulin and collagen fibers, which creates vascular tightening and hypoxia in the central area. This hypoxia damages the centrilobular hepatocytes and the inner hepatic plate, resulting in hepatic dysfunction [60,62]. Non-classical RILD occurs in patients that have an underlying chronic liver disease such as cirrhosis and chronic viral hepatitis, as they have a deregulated liver with jaundice and/or elevated serum transaminase levels. The symptoms appear approximately 3 months after RT, and they may be associated with irreversible damage [59,60,63].

### 4.2. Target Population for Radiation

Most HCC patients do not develop symptoms until the tumor is at a developed stage, resulting in late detection, which makes them not eligible for potentially curative surgical treatments, such as resection and liver transplantation [64,65]. These patients generally benefit most from locoregional therapies such as external beam radiation therapy (EBRT) [66]. In a phase 3 randomized trial, RTOG-1112, patients with advanced HCC treated with stereotactic body radiation therapy (SBRT) plus sorafenib exhibited increased median OS (15.8 vs. 12.3 months; *p* = 0.0554) and median PFS (9.2 vs. 5.5 months) over patients treated with sorafenib alone [67], thus establishing EBRT as standard of care for patients with HCC.

Ionizing radiation causes direct and indirect breaks in double stranded DNA, which damages cells’ ability to carry out DNA replication [68,69]. Liver cancer is moderately to highly sensitive to radiation [70]; in addition, the liver is an organ which has the ability to regenerate normal tissue after radiation-caused damage [71]. Radiotherapy can be categorized into definitive/ablative, palliative, consolidative, and adjuvant [66]. Palliative RT is given to patients with advanced and symptomatic HCC to alleviate symptoms such as pain [72]. Definitive RT refers to treatment in which the patient is given a higher dose of radiation with the goal of eradicating the tumor [73,74]. Consolidative radiation is given after complete response (CR) to systematic therapies, such as chemotherapy, to decrease the risk of reoccurrence and produce favorable outcomes for the patients [75]. Even when all the visible parts of the tumor have been resected in surgical treatment, microscopic residual tumor cells may remain undetected, which may lead to recurrence [76]. A study showed that adjuvant radiation as a post-operation treatment is highly effective for patients who underwent narrow margin hepatectomy. The 5-year OS rate was 72.2%, which significantly exceeds the rates in published comparable reports where adjuvant RT was not used on HCC with a resection margin of <1 cm, such as 46.7% [77] 49.1% [78], and 26.7% [79]. Moreover, the 3-year and 5-year DFS rates were found to be 68.1% and 51.6%, respectively, with no RILD or margin failure [80].

### 4.3. Types of Radiation Therapy

#### 4.3.1. Three-Dimensional Conformal Radiation Therapy

Three-dimensional conformal radiation therapy (3DCRT) is a type of EBRT that uses computed tomography (CT) scans to map out the full tumor along with the surrounding environment in the liver and create a plan to deliver a shaped dose of high-energy photon beams to the target, all while protecting the at-risk adjacent organs [81]. The radiation is made up of multiple photon beams with energies up to 15 MV, as well as non-coplanar beams to ensure better concurrence [82,83]. This technique is mostly used in the palliative setting.

#### 4.3.2. Intensity-Modulated Radiation Therapy

Further development in RT techniques led to the creation of intensity-modulated radiation therapy (IMRT), which further expanded the scope of RT from being a traditionally palliative treatment to a curative one. IMRT can deliver a highly precise and conformal dose of radiation using multiple beams, each with varying intensities [84]. The conformal field in IMRT is divided into multiple subfields to administer a non-uniform distribution. This divide allows for a more precise delivery of radiation, where radiation-resistant parts of the tumor receive higher doses and sensitive areas receive lower doses, using either a cone down or a simultaneous integrated boost or dose painting [85,86]. These improvements are accounted for in a study by Yoon et al. that compared the efficacy of 3DCRT to helical IMRT (h-IMRT) in treating patients with HCC. The local control rate (LCR) for 3DCRT and h-IMRT was 28% and 47% (*p* = 0.007), respectively, and the rate of OS for 3DCRT and h-IMRT was 14% and 33% (*p* < 0.001), respectively [87].

#### 4.3.3. Proton Beam Therapy

Proton beam therapy (PBT) can be better than photon beam therapies at protecting organs at risk (OARs), especially the healthy liver parenchyma, due to the lack of exit dose. In liver tumors, the normal surrounding tissues of liver, biliary ducts, and GI tract are sensitive to radiation and thus are a big limiting factor. As a result, PBT is increasingly being studied as a treatment option for HCC patients [88]. Proton beams, like photon beams, collide with DNA and generate reactive oxygen species (ROS), resulting in DNA damage and cytotoxicity [89]. However, the large and charged nature of protons, as opposed to the uncharged and massless nature of photons, makes proton therapies more efficient at producing DNA damage [90]. Most of the energy in protons is retained until it reaches the tumor site, where the remaining energy is lost over a very short distance. This is what results in the distinctive “Bragg peak”, which forms when the tissue rapidly absorbs the dose. This higher level of conformity and control is what allows the sparing of surrounding healthy tissues and organs [91]. Most institutions have come to the agreement that the relative biological effectiveness (RBE) of PBT is 1.1, when compared to photon beam therapies. This means that a proton dose of 1 Gy is equivalent in effect to a photon dose of 1.1 Gy [92].

#### 4.3.4. Stereotactic Body Radiation Therapy

Stereotactic body radiation therapy (SBRT) uses hypo-fractionated beams of radiation with very high precision to control up to 90% of an HCC tumor region [93]. It makes use of image guidance and minimizes motion to deliver the high doses safely. Compounding the high doses into a few fractions lowers the chance of the tumor repopulating or carrying out DNA repair, making the dose more effective [69]. SBRT has been recognized as an alternative treatment for patients that are not eligible for standard therapies such as surgery or ablation [93,94]. It can make use of either protons or photons, depending on the condition and location of the target. A study that compared photon SBRT using volumetric modulated arc therapy (VMAT) to proton SBRT using intensity-modulated proton therapy (IMPT) in prostate cancer found that VMAT achieves greater conformity and OAR sparing [95]. On the other hand, a different study that also compared proton SBRT to photon SBRT in unresectable HCC found that proton SBRT resulted in greater OS and a decreased risk of RILD [96].

## 5. Rationale for Combination Therapy

### 5.1. Rationale for the Potential Synergy between Radiotherapy and Immunotherapy in HCC

Treatment of HCC patients with either immunotherapy or RT alone presents certain drawbacks. The limitation of treatment with RT alone lies in the property of liver tumors in acquiring radio-resistance post-radiation through the upregulation of PD-L1, which leads to CD8^+^ T cell inhibition and immune evasion [97]. In addition, RT promotes the upregulation of CTLA-4, PD-1, PD-L1, and VEGF [18]. In the case of immunotherapy, it has been reported in pre-clinical and clinical studies that liver metastases derive a decreased benefit from the treatment due to liver tumors’ immunosuppressive microenvironment as liver tumors have been shown to recruit immunosuppressive MDSCs, which suppress T cell function, leading to immunotherapy resistance [13].

The rationale for combining RT with immunotherapy stems from the characteristics of each treatment in overcoming the other’s limitations, thus achieving a synergetic anti-tumor effect. For instance, high-dose liver RT was found to cause a reduction in MDSCs in the peripheral blood and tumor tissues of mouse models transplanted with liver tumors, which proved to be essential in achieving an abscopal effect when the irradiated tumors were inoculated in off-target locations [98]. Similar results were reported in mouse models whereby RT reshaped the liver tumor microenvironment through the decrease in chemokines CCL2, CCL11, and CXCL2, which are responsible for inducing MDSC trafficking in the liver. In addition, RT resulted in higher infiltration and decreased apoptosis of hepatic T cells in the liver tumor microenvironment [13]. A study evaluating the frequency of MDSCs in HCC patients receiving IMRT or 3DCRT reported an average MDSC frequency of 17% prior to radiation, which was decreased to 13% post-radiation [99]. Meanwhile, the addition of PD-1 or PD-L1 blockers allows the restoration of the anti-tumor activity of radiation-induced exhausted CD8^+^ T cells, and the addition of CTLA-4 blockers serves to overcome the RT-induced inhibitory signals on APCs and regulatory T cells. Combination with VEGF blockers would also enhance the immune response through the reduction of infiltrating TAMs and regulatory T cell levels and the activation of CD8^+^ T cells [18]. Another study that stressed the importance of including RT evaluated the health outcomes of 76 patients with HCC, 33 (43.4%) of which were administered a triple therapy of ICI, anti-angiogenic agent, and RT, while 43 (56.6%) received only ICIs and anti-angiogenic agents. The triple therapy group demonstrated a higher ORR of 75.9% vs. 24.1%. Moreover, the addition of RT slowed down the progression of residual liver dysfunction, which incurred a survival benefit in the patients [100].

### 5.2. Pre-Clinical Studies on Combination Therapy in HCC

Several pre-clinical studies have reported on the synergetic anti-tumor responses induced by the combination of RT with PD-1 or PD-L1 blockers. In a study conducted by Kim et al., in which murine HCC models were given a single dose of 10 Gy RT followed by four injections of anti-PD-L1 administered in 3-day intervals post-RT or either treatment alone, the combination arm revealed significant tumor growth suppression compared to the other groups and a significantly higher 7-week survival rate (90% in the combination group vs. 30% in the radiation group vs. 0% in the anti-PD-L1 group; *p* < 0.001) [101]. In another study, orthotopic HCC murine models given anti-PD-1 antibodies with concurrent 30 Gy SBRT in three fractions were found to have higher levels of CD8^+^ T cell infiltration at the tumor site, significantly delayed tumor growth, and improved survival [102]. Similarly, mice with subcutaneous and liver tumors treated with RT, anti-PD-L1, or the combination of both were found to exhibit significantly higher T cell infiltration within the subcutaneous tumor in the combination group, while T cell levels did not increase in the anti-PD-L1 group, and RT alone was not sufficient to modulate the amount of T cells by itself. In addition, the combination group displayed improved prolonged survival and regression of the subcutaneous and liver tumors [13]. Finally, a pre-clinical study looking to assess the abscopal effects in the tumors of syngeneic HCC mouse models given RT with or without anti-PD-1 antibodies found significantly an enhanced abscopal effect within the irradiated and non-irradiated tumors in the combination arm, in addition to higher infiltration of activated CD8^+^ T cells [103].

### 5.3. Timing of Radiotherapy and Immunotherapy Treatment

Although ICIs can be given prior to, concurrently, or after radiation, there is currently no clinical consensus on the optimal treatment sequence for the combination. A pre-clinical study looking to compare three combination regimens in colorectal, breast, and melanoma tumor mouse models demonstrated that administering anti-PD-L1 concurrently with RT achieved long-term tumor control rather than when it was administered 7 days post-RT. In addition, the authors reported peak PD-L1 expression on tumor cells 3 days post-RT with a significant decline as of day 7 [104]. Moreover, a mathematical model simulating the effect of combining RT with ICIs, based on trial data of HCC patients treated with durvalumab (anti-PD-L1), demonstrated that maximal response is observed when RT and ICIs are given concurrently and that there is a significant reduction in response when the timing between the end of RT and the start of ICIs increases [105]. In another pre-clinical study, anti-CTLA-4 was administered in mammary tumor mouse models either 7 days before, 1 day after, or 5 days after RT and the best tumor control was achieved when anti-CTLA-4 was given prior to RT. The authors propose that this is partly due to anti-CTLA-4’s ability to deplete regulatory T cells [106]. These studies suggest that the optimal timing for the delivery of ICIs with radiation depends on the mechanism of action of the chosen ICI. Further patient studies on the ideal treatment sequence for ICIs with RT in HCC patients are required to confirm these findings in clinical settings.

## 6. Current Trials

### 6.1. Clinical Trials for RT Plus ICIs in HCC Patients

Several clinical trials have investigated the combination of RT and ICIs as a treatment plan for patients with HCC. Many of those trials differ by the type of ICI or RT used and the time at which each type of medication was administered (Table 2). One such study reported on HCC patients treated with the combination of neoadjuvant or adjuvant atezolizumab plus bevacizumab and/or concurrent atezolizumab, followed by IMRT, PBT, or 3DCRT. The authors concluded that the combined treatment plan is safe and does not cause liver decompensation or acute hepatitis since liver enzyme levels remained stable for up to 2 months after RT. Moreover, patients receiving the combined therapy (atezolizumab plus bevacizumab as neoadjuvant, concurrent, or adjuvant therapy) had faster recovery times for absolute lymphocyte counts (ALCs) compared to patients who had only received RT [107].

Other clinical trials studied administering RT prior to ICIs. A phase 1 clinical trial looking at 13 advanced or unresectable HCC patients treated with SBRT, followed by nivolumab and/or ipilimumab 2 weeks later, reported dose-limiting toxicities occurring within 6 months in 2 patients (1 in the nivolumab group and 1 in the nivolumab plus ipilimumab group). Moreover, the combined use of nivolumab plus ipilimumab produced better outcomes than the use of nivolumab alone (ORR: 57% vs. 0%, median OS: 41.6 vs. 4.7 months, median PFS: 11.6 vs. 2.7 months, respectively; *p* < 0.05) [108]. A case series evaluated five patients with unresectable HCC treated with SBRT followed by nivolumab monotherapy two weeks later, four of which received transarterial chemoembolization (TACE) 4 weeks prior to RT. The authors reported a median PFS of 14.9 months, an ORR and LCR of 100%, and no incidence of classical RILD [109]. Another study investigated the health outcomes of unresectable HCC patients who were treated with upfront RT, including those receiving concurrent nivolumab and RT or receiving RT at a median of 3.8 months before nivolumab was administered, or salvage RT at 4 weeks after initiation of nivolumab. The ORR was higher in the upfront RT arm (34.6%) compared to the salvage RT arm (11.1%) and no significant differences in ORR were observed within the upfront RT group between patients receiving concurrent or neoadjuvant RT (38% vs. 33%, respectively) [110]. A similar retrospective study that looked into patients with advanced HCC who were treated with previous or concurrent RT with nivolumab focused on the Child–Pugh (CP) and albumin–bilirubin (ALBI) scores. The mean increase in CP and ALBI at 1 month was 0.46 (95% CI 0.22–0.60) and 0.10 (95% CI 0.02–0.18), respectively, and 0.08 (95% CI −0.40–0.56) and 0.02 (95% CI −0.32–0.36) at 6 months, respectively. In addition, patients receiving prior RT presented with a higher rate of grade 3+ toxicities than those receiving concurrent RT (21.3% vs. 8.1%, respectively; *p* = 0.035) [111]. Lastly, a clinical trial compared the effectiveness of administering SBRT followed by nivolumab to TACE only. The SBRT–nivolumab group had significantly higher 12- and 24-month PFS (93.3% vs. 16.7% and 77.8% vs. 2.1%, respectively; *p* < 0.001) and OS (93.8% vs. 31.3% and 80.4% vs. 8.3%, respectively; *p* < 0.001) than those of the TACE group. Moreover, the ORR for the SBRT–nivolumab arm was 87.5%, which was significantly higher than the 16.7% ORR of those receiving TACE (*p* < 0.001) [112].

Other clinical trials studied the health outcomes of treating HCC patients with RT and ICIs concurrently. A phase 2 clinical trial investigating the health outcomes of unresectable HCC patients who were treated with palliative SBRT and camrelizumab simultaneously reported an ORR of 52.4%, a PFS of 5.8 months, and OS of 14.2 months. In addition, no patient developed grade 4/5 treatment-related adverse events (TRAEs) [113]. In a retrospective study looking at advanced HCC patients who had received anti-PD1/PD-L1 followed by PBT within a month of the ICI, 12 patients were given a combination of ICIs with tyrosine kinase inhibitors, anti-VEGF, or other ICIs (4 with nivolumab plus sorafenib or lenvatinib; 6 with bevacizumab plus nivolumab or atezolizumab; and 2 with nivolumab plus ipilimumab), and 17 patients received anti-PD-1 monotherapy (15 nivolumab and 2 pembrolizumab). PBT was delivered with curative intent or palliative control in 13 and 16 patients, respectively. Patients receiving curative-intent PBT achieved a higher ORR (61.5% vs. 43.8%; *p* = 0.340) and medium overall PFS (27.2 months vs. 15.9 months; *p* = 0.118) compared to patients who were given palliative-intent PBT [114].

Table 2 provides a summary of the clinical studies mentioned in this review, which involve the combination treatment of RT and ICIs with or without other agents in HCC patients.

### 6.2. Clinical Trials for RT Plus ICIs Plus TKIs

Many studies looked into a triple therapy approach to treat HCC patients, where combinations of RT, ICIs, and tyrosine kinase inhibitors (TKIs) are used. A study looked at HCC patients whose treatment consisted of an anti-PD-1 being administered once every three weeks, followed by IMRT a week after, and sorafenib daily. The results for the ORR, mOS, and median progression-free survival (mPFS) of the group receiving the triple therapy were greater than those of the control group who did not receive RT (ORR: 42.6% vs. 24.5%; *p* = 0.013; mOS: 20.1 vs. 13.3 months; *p* = 0.009; mPFS: 8.7 vs. 5.4 months; *p* = 0.001) [115]. A different study demonstrated that the combination of camrelizumab and sorafenib, followed by TACE two weeks later and SBRT within one month, was an effective technique for down-staging tumors in advanced HCC patients with portal vein tumor thrombus (PVTT). Out of the twelve enrolled patients, four (33.3%) demonstrated successful down-staging. Moreover, the ORR and disease control rate (DCR) were 41.7% and 50.0%, respectively, while the median PFS was 15.7 months. These results show that this combination is viable for treating HCC [116].

The addition of TKIs to ICIs and radiation was also documented when the radiation preceded the ICI. Patients with unresectable HCC were given SBRT, followed by toripalimab and anlotinib, and achieved an mPFS of 7.4 months, an ORR of 15%, and a DCR of 50.0% [117].

### 6.3. Ongoing Trials

There are numerous ongoing clinical trials looking into the combined use of RT and ICIs (Table 3). A few examples include a phase 2 trial that is looking to test the combination of tremelimumab and durvalumab, followed by radiation delivered during the second cycle of the ICIs in patients with HCC and biliary tract cancer (NCT03482102). Another phase 2 trial is investigating the effect of administering nivolumab every two weeks, followed by EBRT 2–7 days after the first dose of nivolumab (NCT04611165).

For cases in which RT precedes ICIs, a pilot study will be investigating the health outcomes of patients with resectable HCC treated with SBRT followed by atezolizumab plus bevacizumab (NCT04857684). Two ongoing trials are planning to evaluate treatment of HCC patients with SBRT, followed by sintilimab 4–6 weeks later in comparison with SBRT alone (NCT04167293) or sintilimab alone (NCT04547452).

Lastly, for the case of concurrent treatment with RT and ICIs, a phase 1 study is planning to treat resectable HCC patients with tislelizumab plus SBRT (NCT05185531). Another trial is also looking to assess the outcome of combined ICI plus TKI plus RT through the administration of sintilimab, RT, and lenvatinib in resectable HCC patients with PVTT (NCT05225116).

## 7. Conclusions

Hepatocellular carcinoma is an aggressive type of primary liver cancer with the ability to alter the microenvironment of the liver, decreasing its immune response and facilitating tumor proliferation. As a result, the immunosuppressive effect of HCC diminishes the efficacy of immunotherapeutic drugs. Radiotherapy has been shown to reverse some of these effects, by increasing the infiltration of T cells in the liver and diminishing T cell apoptosis, all while increasing tumor cell death. This reshaping of the immune microenvironment may restore the efficacy of immunotherapy drugs, such as ICIs. However, there remain some drawbacks to RT as a tumor can become radio-resistant through the upregulation of PD-L1. Meanwhile, ICIs can restore anti-tumor immunity by targeting those markers, thereby potentially achieving a synergetic effect when combined with RT. The possibility of adding anti-VEGF drugs to the RT + ICI treatment regimen requires further exploration but is promising in part because of the normalization of tumor vascularization. More comparative trials are required to fully understand the implications of this triple combination as well as the timing and sequencing of each treatment. Lastly, to achieve the best possible outcomes, more research needs to be carried out to determine which type of RT works best in combination with which ICIs.

## Figures and Tables

**Table 1:** Role of several immune checkpoint inhibitors used to treat hepatocellular carcinoma.

Immune Checkpoint Inhibitors	Target	Timing and Location in Immunity Cycle [45]	Mechanism of Action [41,45]
Ipilimumab	CTLA-4	T cell priming phase in lymph nodes	Anti-CTLA-4 binds to the CTLA-4 receptor on CD4^+^, CD8^+^, and regulatory T cells allowing T cell costimulation and activation
Tremelimumab			
Nivolumab	PD-1	T cell effector phase in peripheral tissues	Anti-PD-1 binds to the PD-1 receptor on T cells, B cells, and myeloid cells, allowing immune detection of tumor cells
Pembrolizumab			
Sintilimab			
Tislelizumab			
Camrelizumab			
Toripalimab			
Atezolizumab	PD-L1	T cell effector phase in peripheral tissues	Anti-PD-L1 binds to PD-L1 on APCs, tumor, and other immune cells, allowing immune detection of tumor cells
Durvalumab			

CTLA-4: Cytotoxic T lymphocyte-associated protein 4; PD-1: programmed cell death receptor 1; PD-L1: programmed cell death ligand 1; APCs: antigen-presenting cells.

**Table 2:** Studies on the combination of RT with ICIs and/or TKIs and/or locoregional therapies in HCC patients.

Paper	Treatment Arms	Immunotherapeutic Drugs	RT Type	Combination Treatment Sequence	Primary Endpoint
Manzar et al. [107]	RT + anti-PD-L1 + anti-VEGF	Atezolizumab, bevacizumab	IMRT or PBT or 3DCRT	Neoadjuvant or adjuvant atezolizumab plus bevacizumab and/or concurrent atezolizumab	mOS: 16.1 months
Juloori et al. [108]	RT + anti-PD-1 + anti-CTLA-4 vs. RT + anti-PD-1	Nivolumab, ipilimumab	SBRT	Neoadjuvant RT 2 weeks prior to ICIs	Dose-limiting toxicity occurring within 6 months of RT: 1 patient vs. 1 patient
Chiang et al. [109]	TACE + RT + anti-PD-1 (4 patients) and RT + anti-PD-1 (1 patient)	Nivolumab	SBRT	TACE: 4 weeks prior to RT + neoadjuvant RT 2 weeks prior to ICI	mPFS: 14.9 months 1-year LCR: 100% 1-year OS rate: 100%
Smith et al. [110]	Upfront RT + anti-PD-1 vs. Salvage RT + anti-PD-1	Nivolumab	SBRT (in 47% of cases)	Concurrent or neoadjuvant RT vs. adjuvant RT	ORR: 34.6% vs. 11.1%
Smith et al. [111]	RT + anti-PD-1	Nivolumab	SBRT (in 63.2% of cases)	Neoadjuvant vs. concurrent RT	Rate of grade 3+ toxicities: 21.3% vs. 8.1% of patients (*p* = 0.035)
Chiang et al. [112]	RT + anti-PD-1 vs. TACE	Nivolumab	SBRT	Neoadjuvant RT 2 weeks prior to ICI	PFS (12 months): 93.3% vs. 16.7% PFS (24 months): 77.8% vs. 2.1% (*p* < 0.001)
Li et al. [113]	RT + anti-PD-1	Camrelizumab	SBRT	Concurrent RT	ORR: 52.4% 23.8% of patients had grade 3 TRAEs No patient had grade 4–5 TRAEs
Su et al. [114]	RT (curative) + anti-PD-1/PD-L1 monotherapy or combination with anti-VEGF or mTKI or anti-CTLA-4 vs. RT (palliative) + anti-PD-1/PD-L1 monotherapy or combination with anti-VEGF or mTKI or anti-CTLA-4	Nivolumab or pembrolizumab monotherapy; or nivolumab plus sorafenib or lenvatinib; or bevacizumab plus nivolumab or atezolizumab; or nivolumab plus ipilimumab	PBT	Concurrent RT	1-year PBT infield tumor control: 90.5% vs. 70.8% 1-year PBT outfield tumor control: 90.9% vs. 69.2% OR: 61.5% vs. 43.8% mPFS: 27.2 vs. 15.9 months
Su et al. [115]	RT + anti-PD-1 + anti-VEGF vs. anti-PD-1 + anti-VEGF	Unspecified	IMRT	mTKI daily, concurrent RT (within 7 days of 1 at ICI cycle)	ORR: 40% vs. 25% (*p* = 0.152) mPFS: 8.7 vs. 5.4 months (*p* = 0.013) mOS: 18.5 vs. 12.6 months (*p* = 0.043)
Huang et al. [116]	RT + anti-PD-1 + mTKI + TACE	Camrelizumab, sorafenib	SBRT	mTKI daily and concurrently with ICI, followed by TACE within 2 weeks and RT within 1 month	OR rate: 41.7% DCR: 50% mPFS: 15.7 months mOS: not reached
Chen et al. [117]	RT + anti-PD-1 + anti-VEGF	Toripalimab, anlotinib	SBRT	RT given in 3 days, followed after a 1-day interval by ICI and anti-VEGF	PFS: 7.4 months

RT: radiation therapy; ICI: immune checkpoint inhibitor; CTLA-4: cytotoxic T lymphocyte-associated protein 4; PD-1: programmed cell death receptor 1; PD-L1: programmed cell death ligand 1; VEGF: vascular endothelial growth factor; IMRT: intensity-modulated radiation therapy; PBT: proton beam therapy; 3DCRT: three-dimensional conformal radiation therapy; SBRT: stereotactic body radiation therapy; TACE: transarterial chemoembolization; mTKI: multityrosine kinase inhibitor; ORR: objective response rate; OR: overall response; (m)OS: (median) overall survival; DCR: disease control rate; LCR: local control rate; (m)PFS: (median) progression-free survival; TRAEs: treatment-related adverse events.

**Table 3:** Ongoing clinical trials on the combination of RT with ICIs and/or TKIs in HCC patients.

Ongoing Trial	Treatment Arms	Immunotherapeutic Drug	RT Type	Combination Treatment Sequence	Dosage	Primary Endpoint
NCT03482102	RT+ anti-CTLA-4 + anti-PD-L1	Tremelimumab, durvalumab	NA	Tremelimumab plus durvalumab: up to 4 doses/cycles Durvalumab monotherapy: started on week 16 for up to 8 months given every 4 weeks RT: given only during cycle 2	NA	Best OR rate
NCT04611165	RT + anti-PD-1	Nivolumab	EBRT	Nivolumab: every 2 weeks EBRT: 2–7 days after first dose of nivolumab	PTV1: 30–50 Gy/10 fx (5 Gy/fx) PTV2: 30 Gy/10 fx (3 Gy/fx) Nivolumab: 3 mg/kg	PFS
NCT04857684	RT + anti-PD-L1 + anti-VEGF	Atezolizumab, bevacizumab	SBRT	Neoadjuvant SBRT Atezolizumab: given on day 1 of two 3-week cycles Bevacizumab: 1 time weekly for two 3-week cycles	NA	Proportion of patients with grade 3-4 TRAEs
NCT04167293	RT + anti-PD-1 vs. RT	Sintilimab	SBRT via VMAT	SBRT (1–2 weeks) followed by sintilimab 4–6 weeks later (every 3 weeks for up to 1 year)	RT: 30–54 Gy/3–6 fx (prescribed dose) Sintilimab: 200 mg	24-week PFS rate
NCT04547452	RT + anti-PD-1 vs. anti-PD-1	Sintilimab	SBRT via VMAT	SBRT (1–2 weeks) followed by sintilimab 4–6 weeks later (every 3 weeks for up to 1 year)	RT: 30–54 Gy/3–6 fx (prescribed dose) Sintilimab: 200 mg	24-week PFS rate
NCT05185531	RT + anti-PD-1	Tislelizumab	SBRT	Concurrent RT (days 1, 3, and 5) plus tislelizumab (days 1 and 22)	RT: 8 Gy/3 fx	Delay to surgery, ORR, pathologic response rate, incidence of TRAEs
NCT05225116	RT + anti-PD-1 + mTKI	Sintilimab, lenvatinib	NA	RT: given within 2 weeks Sintilimab: every 3 weeks Lenvatinib: daily starting from day 1	RT: 300 cGy/10 fx Sintilimab: 200 mg Lenvatinib: 8 mg/day ≤ 60 kg or 12 mg/day ≥ 60 kg	Incidence of grade ≥ 3 TRAEs, number of patients who completed pre-op treatment and proceeded to surgery

RT: radiation therapy; CTLA-4: cytotoxic T lymphocyte-associated protein 4; PD-1: programmed cell death receptor 1; PD-L1: programmed cell death ligand 1; VEGF: vascular endothelial growth factor; SBRT: stereotactic body radiation therapy; VMAT: volumetric modulated arc therapy; fx: fraction; mTKI: multityrosine kinase inhibitor; ORR: objective response rate; OR: overall response; PFS: progression-free survival; TRAEs: treatment-related adverse events.

## References

[1] Sung H., Ferlay J., Siegel R.L., Laversanne M., Soerjomataram I., Jemal A., Bray F. (2021). Global Cancer Statistics 2020: GLOBOCAN Estimates of Incidence and Mortality Worldwide for 36 Cancers in 185 Countries. CA Cancer J. Clin..

[2] Llovet J.M., Kelley R.K., Villanueva A., Singal A.G., Pikarsky E., Roayaie S., Lencioni R., Koike K., Zucman-Rossi J., Finn R.S. (2021). Hepatocellular carcinoma. Nat. Rev. Dis. Primers.

[3] Villanueva A. (2019). Hepatocellular Carcinoma. N. Engl. J. Med..

[4] Dhanasekaran R., Bandoh S., Roberts L.R. (2016). Molecular pathogenesis of hepatocellular carcinoma and impact of therapeutic advances. F1000Res.

[5] Asafo-Agyei K.O., Samant H. (2023). Hepatocellular Carcinoma. StatPearls.

[6] Mittal S., El-Serag H.B. (2013). Epidemiology of hepatocellular carcinoma: Consider the population. J. Clin. Gastroenterol..

[7] Benson A.B., D’Angelica M.I., Abbott D.E., Anaya D.A., Anders R., Are C., Bachini M., Borad M., Brown D., Burgoyne A. (2021). Hepatobiliary Cancers, Version 2.2021, NCCN Clinical Practice Guidelines in Oncology. J. Natl. Compr. Cancer Netw..

[8] Inchingolo R., Posa A., Mariappan M., Spiliopoulos S. (2019). Locoregional treatments for hepatocellular carcinoma: Current evidence and future directions. World J. Gastroenterol..

[9] Yang J.D., Hainaut P., Gores G.J., Amadou A., Plymoth A., Roberts L.R. (2019). A global view of hepatocellular carcinoma: Trends, risk, prevention and management. Nat. Rev. Gastroenterol. Hepatol..

[10] Galle P.R., Dufour J.F., Peck-Radosavljevic M., Trojan J., Vogel A. (2021). Systemic therapy of advanced hepatocellular carcinoma. Future Oncol..

[11] Finn R.S., Qin S., Ikeda M., Galle P.R., Ducreux M., Kim T.-Y., Kudo M., Breder V., Merle P., Kaseb A.O. (2020). Atezolizumab plus bevacizumab in unresectable hepatocellular carcinoma. N. Engl. J. Med..

[12] Abou-Alfa G.K., Lau G., Kudo M., Chan S.L., Kelley R.K., Furuse J., Sukeepaisarnjaroen W., Kang Y.-K., Van Dao T., De Toni E.N. (2022). Tremelimumab plus durvalumab in unresectable hepatocellular carcinoma. NEJM Evid..

[13] Yu J., Green M.D., Li S., Sun Y., Journey S.N., Choi J.E., Rizvi S.M., Qin A., Waninger J.J., Lang X. (2021). Liver metastasis restrains immunotherapy efficacy via macrophage-mediated T cell elimination. Nat. Med..

[14] Aravalli R.N. (2013). Role of innate immunity in the development of hepatocellular carcinoma. World J. Gastroenterol..

[15] Sachdeva M., Chawla Y.K., Arora S.K. (2015). Immunology of hepatocellular carcinoma. World J. Hepatol..

[16] Spitz D.R., Azzam E.I., Li J.J., Gius D. (2004). Metabolic oxidation/reduction reactions and cellular responses to ionizing radiation: A unifying concept in stress response biology. Cancer Metastasis Rev..

[17] Chen L., Zhang R., Lin Z., Tan Q., Huang Z., Liang B. (2023). Radiation therapy in the era of immune treatment for hepatocellular carcinoma. Front. Immunol..

[18] Lee Y.H., Tai D., Yip C., Choo S.P., Chew V. (2020). Combinational Immunotherapy for Hepatocellular Carcinoma: Radiotherapy, Immune Checkpoint Blockade and Beyond. Front. Immunol..

[19] Liu Y., Dong Y., Kong L., Shi F., Zhu H., Yu J. (2018). Abscopal effect of radiotherapy combined with immune checkpoint inhibitors. J. Hematol. Oncol..

[20] Bieghs V., Trautwein C. (2013). The innate immune response during liver inflammation and metabolic disease. Trends Immunol..

[21] Racanelli V., Rehermann B. (2006). The liver as an immunological organ. Hepatology.

[22] De Re V., Rossetto A., Rosignoli A., Muraro E., Racanelli V., Tornesello M.L., Zompicchiatti A., Uzzau A. (2022). Hepatocellular Carcinoma Intrinsic Cell Death Regulates Immune Response and Prognosis. Front. Oncol..

[23] Llovet J.M., Castet F., Heikenwalder M., Maini M.K., Mazzaferro V., Pinato D.J., Pikarsky E., Zhu A.X., Finn R.S. (2022). Immunotherapies for hepatocellular carcinoma. Nat. Rev. Clin. Oncol..

[24] Fu J., Xu D., Liu Z., Shi M., Zhao P., Fu B., Zhang Z., Yang H., Zhang H., Zhou C. (2007). Increased regulatory T cells correlate with CD8 T-cell impairment and poor survival in hepatocellular carcinoma patients. Gastroenterology.

[25] Hoechst B., Ormandy L.A., Ballmaier M., Lehner F., Krüger C., Manns M.P., Greten T.F., Korangy F. (2008). A new population of myeloid-derived suppressor cells in hepatocellular carcinoma patients induces CD4^+^ CD25^+^ Foxp3^+^ T cells. Gastroenterology.

[26] Ren Z., Xu J., Bai Y., Xu A., Cang S., Du C., Li Q., Lu Y., Chen Y., Guo Y. (2021). Sintilimab plus a bevacizumab biosimilar (IBI305) versus sorafenib in unresectable hepatocellular carcinoma (ORIENT-32): A randomised, open-label, phase 2–3 study. Lancet Oncol..

[27] Mode D. AstraZeneca: Imfinzi Plus Imjudo Demonstrated Sustained Overall Survival Benefit in Advanced Liver Cancer with an Unprecedented One in Four Patients Alive at Four Years in HIMALAYA Phase III Trial. https://mfn.se/cis/a/astrazeneca/astrazeneca-imfinzi-plus-imjudo-demonstrated-sustained-overall-survival-benefit-in-advanced-liver-cancer-with-an-unprecedented-one-in-four-patients-alive-at-four-years-in-himalaya-phase-iii-trial-4799270e.

[28] Yau T., Park J.W., Finn R.S., Cheng A.L., Mathurin P., Edeline J., Kudo M., Harding J.J., Merle P., Rosmorduc O. (2022). Nivolumab versus sorafenib in advanced hepatocellular carcinoma (CheckMate 459): A randomised, multicentre, open-label, phase 3 trial. Lancet Oncol..

[29] Yau T., Kang Y.K., Kim T.Y., El-Khoueiry A.B., Santoro A., Sangro B., Melero I., Kudo M., Hou M.M., Matilla A. (2020). Efficacy and Safety of Nivolumab Plus Ipilimumab in Patients With Advanced Hepatocellular Carcinoma Previously Treated With Sorafenib: The CheckMate 040 Randomized Clinical Trial. JAMA Oncol..

[30] Zhu A.X., Finn R.S., Edeline J., Cattan S., Ogasawara S., Palmer D., Verslype C., Zagonel V., Fartoux L., Vogel A. (2018). Pembrolizumab in patients with advanced hepatocellular carcinoma previously treated with sorafenib (KEYNOTE-224): A non-randomised, open-label phase 2 trial. Lancet Oncol..

[31] Zhu A.X., Kang Y.K., Yen C.J., Finn R.S., Galle P.R., Llovet J.M., Assenat E., Brandi G., Pracht M., Lim H.Y. (2019). Ramucirumab after sorafenib in patients with advanced hepatocellular carcinoma and increased α-fetoprotein concentrations (REACH-2): A randomised, double-blind, placebo-controlled, phase 3 trial. Lancet Oncol..

[32] Psilopatis I., Damaskos C., Garmpi A., Sarantis P., Koustas E., Antoniou E.A., Dimitroulis D., Kouraklis G., Karamouzis M.V., Vrettou K. (2023). FDA-Approved Monoclonal Antibodies for Unresectable Hepatocellular Carcinoma: What Do We Know So Far?. Int. J. Mol. Sci..

[33] Kelley R.K., Mollon P., Blanc J.F., Daniele B., Yau T., Cheng A.L., Valcheva V., Marteau F., Guerra I., Abou-Alfa G.K. (2020). Comparative Efficacy of Cabozantinib and Regorafenib for Advanced Hepatocellular Carcinoma. Adv. Ther..

[34] Ducreux M., Abou-Alfa G., Ren Z., Edeline J., Li Z., Assenat E., Rimassa L., Blanc J., Ross P., Fang W. (2021). O-1 Results from a global phase 2 study of tislelizumab, an investigational PD-1 antibody, in patients with unresectable hepatocellular carcinoma. Ann. Oncol..

[35] Qin S., Ren Z., Meng Z., Chen Z., Chai X., Xiong J., Bai Y., Yang L., Zhu H., Fang W. (2020). Camrelizumab in patients with previously treated advanced hepatocellular carcinoma: A multicentre, open-label, parallel-group, randomised, phase 2 trial. Lancet Oncol..

[36] Qin S., Chan L., Gu S., Bai Y., Ren Z., Lin X., Chen Z., Jia W., Jin Y., Guo Y. (2022). LBA35 Camrelizumab (C) plus rivoceranib (R) vs. sorafenib (S) as first-line therapy for unresectable hepatocellular carcinoma (uHCC): A randomized, phase III trial. Ann. Oncol..

[37] Zhang C.S., Zeng Z.M., Zhuo M.Y., Luo J.R., Zhuang X.H., Xu J.N., Zeng J., Ma J., Lin H.F. (2023). Anlotinib Combined With Toripalimab as First-Line Therapy for Unresectable Hepatocellular Carcinoma: A Prospective, Multicenter, Phase II Study. Oncologist.

[38] Zhang Y., Zheng J. (2020). Functions of Immune Checkpoint Molecules Beyond Immune Evasion. Adv. Exp. Med. Biol..

[39] Pennock G.K., Chow L.Q. (2015). The evolving role of immune checkpoint inhibitors in cancer treatment. Oncologist.

[40] Saad P., Kasi A. (2020). Ipilimumab.

[41] Walker L.S., Sansom D.M. (2011). The emerging role of CTLA4 as a cell-extrinsic regulator of T cell responses. Nat. Rev. Immunol..

[42] Zhang H., Dai Z., Wu W., Wang Z., Zhang N., Zhang L., Zeng W.J., Liu Z., Cheng Q. (2021). Regulatory mechanisms of immune checkpoints PD-L1 and CTLA-4 in cancer. J. Exp. Clin. Cancer Res..

[43] Kudo M. (2019). Scientific rationale for combination immunotherapy of hepatocellular carcinoma with anti-PD-1/PD-L1 and anti-CTLA-4 antibodies. Liver Cancer.

[44] Keam S.J. (2023). Tremelimumab: First Approval. Drugs.

[45] Buchbinder E.I., Desai A. (2016). CTLA-4 and PD-1 Pathways: Similarities, Differences, and Implications of Their Inhibition. Am. J. Clin. Oncol..

[46] Yokosuka T., Takamatsu M., Kobayashi-Imanishi W., Hashimoto-Tane A., Azuma M., Saito T. (2012). Programmed cell death 1 forms negative costimulatory microclusters that directly inhibit T cell receptor signaling by recruiting phosphatase SHP2. J. Exp. Med..

[47] Kudo M. (2020). Scientific Rationale for Combined Immunotherapy with PD-1/PD-L1 Antibodies and VEGF Inhibitors in Advanced Hepatocellular Carcinoma. Cancers.

[48] Merle P., Blanc J.F., Edeline J., Le Malicot K., Allaire M., Assenat E., Guarssifi M., Bouattour M., Péron J.M., Laurent-Puig P. (2023). Ipilimumab with atezolizumab-bevacizumab in patients with advanced hepatocellular carcinoma: The PRODIGE 81-FFCD 2101-TRIPLET-HCC trial. Dig. Liver Dis..

[49] Hönscheid P., Datta K., Muders M.H. (2014). Autophagy: Detection, regulation and its role in cancer and therapy response. Int. J. Radiat. Biol..

[50] Balkwill F., Mantovani A. (2001). Inflammation and cancer: Back to Virchow?. Lancet.

[51] Deng L., Liang H., Xu M., Yang X., Burnette B., Arina A., Li X.D., Mauceri H., Beckett M., Darga T. (2014). STING-Dependent Cytosolic DNA Sensing Promotes Radiation-Induced Type I Interferon-Dependent Antitumor Immunity in Immunogenic Tumors. Immunity.

[52] Burnette B.C., Liang H., Lee Y., Chlewicki L., Khodarev N.N., Weichselbaum R.R., Fu Y.X., Auh S.L. (2011). The efficacy of radiotherapy relies upon induction of type i interferon-dependent innate and adaptive immunity. Cancer Res..

[53] Gresser I., Belardelli F., Maury C., Maunoury M.T., Tovey M.G. (1983). Injection of mice with antibody to interferon enhances the growth of transplantable murine tumors. J. Exp. Med..

[54] Chakraborty M., Abrams S.I., Camphausen K., Liu K., Scott T., Coleman C.N., Hodge J.W. (2003). Irradiation of tumor cells up-regulates Fas and enhances CTL lytic activity and CTL adoptive immunotherapy. J. Immunol..

[55] Lugade A.A., Moran J.P., Gerber S.A., Rose R.C., Frelinger J.G., Lord E.M. (2005). Local radiation therapy of B16 melanoma tumors increases the generation of tumor antigen-specific effector cells that traffic to the tumor. J. Immunol..

[56] Reynders K., Illidge T., Siva S., Chang J.Y., De Ruysscher D. (2015). The abscopal effect of local radiotherapy: Using immunotherapy to make a rare event clinically relevant. Cancer Treat. Rev..

[57] Krysko D.V., Garg A.D., Kaczmarek A., Krysko O., Agostinis P., Vandenabeele P. (2012). Immunogenic cell death and DAMPs in cancer therapy. Nat. Rev. Cancer.

[58] Lee I.J., Seong J., Shim S.J., Han K.H. (2009). Radiotherapeutic parameters predictive of liver complications induced by liver tumor radiotherapy. Int. J. Radiat. Oncol. Biol. Phys..

[59] Kim J., Jung Y. (2017). Radiation-induced liver disease: Current understanding and future perspectives. Exp. Mol. Med..

[60] Guha C., Kavanagh B.D. (2011). Hepatic radiation toxicity: Avoidance and amelioration. Semin. Radiat. Oncol..

[61] Lawrence T.S., Robertson J.M., Anscher M.S., Jirtle R.L., Ensminger W.D., Fajardo L.F. (1995). Hepatic toxicity resulting from cancer treatment. Int. J. Radiat. Oncol. Biol. Phys..

[62] Reed G.B., Cox A.J. (1966). The human liver after radiation injury. A form of veno-occlusive disease. Am. J. Pathol..

[63] Guha C., Sharma A., Gupta S., Alfieri A., Gorla G.R., Gagandeep S., Sokhi R., Roy-Chowdhury N., Tanaka K.E., Vikram B. (1999). Amelioration of radiation-induced liver damage in partially hepatectomized rats by hepatocyte transplantation. Cancer Res..

[64] Wu M.C. (2008). Progress in diagnosis and treatment of primary liver cancer. Zhongguo Yi Xue Ke Xue Yuan Xue Bao.

[65] Apisarnthanarax S., Barry A., Cao M., Czito B., DeMatteo R., Drinane M., Hallemeier C.L., Koay E.J., Lasley F., Meyer J. (2022). External Beam Radiation Therapy for Primary Liver Cancers: An ASTRO Clinical Practice Guideline. Pract. Radiat. Oncol..

[66] Rim C.H., Seong J. (2016). Application of radiotherapy for hepatocellular carcinoma in current clinical practice guidelines. Radiat. Oncol. J..

[67] Dawson L., Winter K., Knox J., Zhu A., Krishnan S., Guha C., Kachnic L., Gillin M., Hong T., Craig T. (2022). NRG/RTOG 1112: Randomized Phase III Study of Sorafenib vs. Stereotactic Body Radiation Therapy (SBRT) Followed by Sorafenib in Hepatocellular Carcinoma (HCC)(NCT01730937). Int. J. Radiat. Oncol. Biol. Phys..

[68] Kalogeridi M.A., Zygogianni A., Kyrgias G., Kouvaris J., Chatziioannou S., Kelekis N., Kouloulias V. (2015). Role of radiotherapy in the management of hepatocellular carcinoma: A systematic review. World J. Hepatol..

[69] Bang A., Dawson L.A. (2019). Radiotherapy for HCC: Ready for prime time?. JHEP Rep..

[70] Emami B., Lyman J., Brown A., Coia L., Goitein M., Munzenrider J.E., Shank B., Solin L.J., Wesson M. (1991). Tolerance of normal tissue to therapeutic irradiation. Int. J. Radiat. Oncol. Biol. Phys..

[71] Chen W., Chiang C.L., Dawson L.A. (2021). Efficacy and safety of radiotherapy for primary liver cancer. Chin. Clin. Oncol..

[72] Yeung C.S.Y., Chiang C.L., Wong N.S.M., Ha S.K., Tsang K.S., Ho C.H.M., Wang B., Lee V.W.Y., Chan M.K.H., Lee F.A.S. (2020). Palliative Liver Radiotherapy (RT) for Symptomatic Hepatocellular Carcinoma (HCC). Sci. Rep..

[73] Crane C.H., Koay E.J. (2016). Solutions that enable ablative radiotherapy for large liver tumors: Fractionated dose painting, simultaneous integrated protection, motion management, and computed tomography image guidance. Cancer.

[74] Hilal L., Reyngold M., Wu A.J., Araji A., Abou-Alfa G.K., Jarnagin W., Harding J.J., Gambarin M., El Dika I., Brady P. (2021). Ablative radiation therapy for hepatocellular carcinoma is associated with reduced treatment- and tumor-related liver failure and improved survival. J. Gastrointest. Oncol..

[75] Hu C., Deng C., Zou W., Zhang G., Wang J. (2015). The Role of Consolidative Radiotherapy after a Complete Response to Chemotherapy in the Treatment of Diffuse Large B-Cell Lymphoma in the Rituximab Era: Results from a Systematic Review with a Meta-Analysis. Acta Haematol..

[76] Sugarbaker P.H. (1999). Successful management of microscopic residual disease in large bowel cancer. Cancer Chemother. Pharmacol..

[77] Ikai I., Arii S., Kojiro M., Ichida T., Makuuchi M., Matsuyama Y., Nakanuma Y., Okita K., Omata M., Takayasu K. (2004). Reevaluation of prognostic factors for survival after liver resection in patients with hepatocellular carcinoma in a Japanese nationwide survey. Cancer.

[78] Shi M., Guo R.P., Lin X.J., Zhang Y.Q., Chen M.S., Zhang C.Q., Lau W.Y., Li J.Q. (2007). Partial hepatectomy with wide versus narrow resection margin for solitary hepatocellular carcinoma: A prospective randomized trial. Ann. Surg..

[79] Shimada K., Sakamoto Y., Esaki M., Kosuge T. (2008). Role of the width of the surgical margin in a hepatectomy for small hepatocellular carcinomas eligible for percutaneous local ablative therapy. Am. J. Surg..

[80] Chen B., Wu J.X., Cheng S.H., Wang L.M., Rong W.Q., Wu F., Wang S.L., Jin J., Liu Y.P., Song Y.W. (2021). Phase 2 Study of Adjuvant Radiotherapy Following Narrow-Margin Hepatectomy in Patients With HCC. Hepatology.

[81] Soni P.D., Palta M. (2019). Stereotactic Body Radiation Therapy for Hepatocellular Carcinoma: Current State and Future Opportunities. Dig. Dis. Sci..

[82] Kouloulias V., Mosa E., Georgakopoulos J., Platoni K., Brountzos I., Zygogianni A., Antypas C., Kosmidis P., Mystakidou K., Tolia M. (2013). Three-dimensional conformal radiotherapy for hepatocellular carcinoma in patients unfit for resection, ablation, or chemotherapy: A retrospective study. Sci. World J..

[83] Lee J.H., Kim D.H., Ki Y.K., Nam J.H., Heo J., Woo H.Y., Kim D.W., Kim W.T. (2014). Three-dimensional conformal radiotherapy for portal vein tumor thrombosis alone in advanced hepatocellular carcinoma. Radiat. Oncol. J..

[84] Bae S.H., Jang W.I., Park H.C. (2017). Intensity-modulated radiotherapy for hepatocellular carcinoma: Dosimetric and clinical results. Oncotarget.

[85] Galvin J.M., De Neve W. (2007). Intensity modulating and other radiation therapy devices for dose painting. J. Clin. Oncol..

[86] Vlacich G., Stavas M.J., Pendyala P., Chen S.C., Shyr Y., Cmelak A.J. (2017). A comparative analysis between sequential boost and integrated boost intensity-modulated radiation therapy with concurrent chemotherapy for locally-advanced head and neck cancer. Radiat. Oncol..

[87] Yoon H.I., Lee I.J., Han K.H., Seong J. (2014). Improved oncologic outcomes with image-guided intensity-modulated radiation therapy using helical tomotherapy in locally advanced hepatocellular carcinoma. J. Cancer Res. Clin. Oncol..

[88] Yoo G.S., Yu J.I., Park H.C. (2018). Proton therapy for hepatocellular carcinoma: Current knowledges and future perspectives. World J. Gastroenterol..

[89] Alan Mitteer R., Wang Y., Shah J., Gordon S., Fager M., Butter P.P., Jun Kim H., Guardiola-Salmeron C., Carabe-Fernandez A., Fan Y. (2015). Proton beam radiation induces DNA damage and cell apoptosis in glioma stem cells through reactive oxygen species. Sci. Rep..

[90] Durante M., Loeffler J.S. (2010). Charged particles in radiation oncology. Nat. Rev. Clin. Oncol..

[91] Ling T.C., Kang J.I., Bush D.A., Slater J.D., Yang G.Y. (2012). Proton therapy for hepatocellular carcinoma. Chin. J. Cancer Res..

[92] Gerweck L.E., Kozin S.V. (1999). Relative biological effectiveness of proton beams in clinical therapy. Radiother. Oncol..

[93] Sanuki N., Takeda A., Oku Y., Mizuno T., Aoki Y., Eriguchi T., Iwabuchi S., Kunieda E. (2014). Stereotactic body radiotherapy for small hepatocellular carcinoma: A retrospective outcome analysis in 185 patients. Acta Oncol..

[94] Matsuo Y. (2023). Stereotactic Body Radiotherapy for Hepatocellular Carcinoma: A Brief Overview. Curr. Oncol..

[95] Goddard L.C., Brodin N.P., Bodner W.R., Garg M.K., Tomé W.A. (2018). Comparing photon and proton-based hypofractioned SBRT for prostate cancer accounting for robustness and realistic treatment deliverability. Br. J. Radiol..

[96] Sanford N.N., Pursley J., Noe B., Yeap B.Y., Goyal L., Clark J.W., Allen J.N., Blaszkowsky L.S., Ryan D.P., Ferrone C.R. (2019). Protons versus Photons for Unresectable Hepatocellular Carcinoma: Liver Decompensation and Overall Survival. Int. J. Radiat. Oncol. Biol. Phys..

[97] Kreidieh M., Zeidan Y.H., Shamseddine A. (2019). The Combination of Stereotactic Body Radiation Therapy and Immunotherapy in Primary Liver Tumors. J. Oncol..

[98] Chen J., Wang Z., Ding Y., Huang F., Huang W., Lan R., Chen R., Wu B., Fu L., Yang Y. (2020). Hypofractionated Irradiation Suppressed the Off-Target Mouse Hepatocarcinoma Growth by Inhibiting Myeloid-Derived Suppressor Cell-Mediated Immune Suppression. Front. Oncol..

[99] Wang D., An G., Xie S., Yao Y., Feng G. (2016). The clinical and prognostic significance of CD14^+^HLA-DR^−/low^ myeloid-derived suppressor cells in hepatocellular carcinoma patients receiving radiotherapy. Tumour Biol..

[100] Ning C., Zhang X., Wang Y., Yang X., Yang X., Chao J., Xun Z., Xue J., Wang Y., Sun H. (2023). Radiation Therapy With Combination Therapy of Immune Checkpoint Inhibitors and Antiangiogenic Therapy for Hepatocellular Carcinoma. Int. J. Radiat. Oncol. Biol. Phys..

[101] Kim K.-J., Kim J.-H., Lee S.J., Lee E.-J., Shin E.-C., Seong J. (2017). Radiation improves antitumor effect of immune checkpoint inhibitor in murine hepatocellular carcinoma model. Oncotarget.

[102] Friedman D., Baird J.R., Young K.H., Cottam B., Crittenden M.R., Friedman S., Gough M.J., Newell P. (2017). Programmed cell death-1 blockade enhances response to stereotactic radiation in an orthotopic murine model of hepatocellular carcinoma. Hepatol. Res..

[103] Yoo G.S., Ahn W.-G., Kim S.-Y., Kang W., Choi C., Park H.C. (2021). Radiation-induced abscopal effect and its enhancement by programmed cell death 1 blockade in the hepatocellular carcinoma: A murine model study. Clin. Mol. Hepatol..

[104] Dovedi S.J., Adlard A.L., Lipowska-Bhalla G., McKenna C., Jones S., Cheadle E.J., Stratford I.J., Poon E., Morrow M., Stewart R. (2014). Acquired resistance to fractionated radiotherapy can be overcome by concurrent PD-L1 blockade. Cancer Res..

[105] Sung W., Hong T.S., Poznansky M.C., Paganetti H., Grassberger C. (2022). Mathematical modeling to simulate the effect of adding radiation therapy to immunotherapy and application to hepatocellular carcinoma. Int. J. Radiat. Oncol. Biol. Phys..

[106] Young K.H., Baird J.R., Savage T., Cottam B., Friedman D., Bambina S., Messenheimer D.J., Fox B., Newell P., Bahjat K.S. (2016). Optimizing timing of immunotherapy improves control of tumors by hypofractionated radiation therapy. PLoS ONE.

[107] Manzar G.S., De B.S., Abana C.O., Lee S.S., Javle M., Kaseb A.O., Vauthey J.N., Tran Cao H.S., Koong A.C., Smith G.L. (2022). Outcomes and Toxicities of Modern Combined Modality Therapy with Atezolizumab Plus Bevacizumab and Radiation Therapy for Hepatocellular Carcinoma. Cancers.

[108] Juloori A., Katipally R.R., Lemons J.M., Singh A.K., Iyer R., Robbins J.R., George B., Hall W.A., Pitroda S.P., Arif F. (2023). Phase 1 Randomized Trial of Stereotactic Body Radiation Therapy Followed by Nivolumab plus Ipilimumab or Nivolumab Alone in Advanced/Unresectable Hepatocellular Carcinoma. Int. J. Radiat. Oncol. Biol. Phys..

[109] Chiang C.L., Chan A.C.Y., Chiu K.W.H., Kong F.S. (2019). Combined Stereotactic Body Radiotherapy and Checkpoint Inhibition in Unresectable Hepatocellular Carcinoma: A Potential Synergistic Treatment Strategy. Front. Oncol..

[110] Smith W.H., Law A.S., Hulkower M., McGee H.M., Lehrer E.J., Schwartz M., Taouli B., Sung M., Buckstein M. (2020). The effect of radiation therapy on the objective response and outcomes with nivolumab for hepatocellular carcinoma. Acta Oncol..

[111] Smith W., McGee H., Schwartz M., Sung M., Rosenzweig K., Buckstein M. (2019). The safety of nivolumab in combination with prior or concurrent radiation therapy among patients with hepatocellular carcinoma. Int. J. Radiat. Oncol. Biol. Phys..

[112] Chiang C.L., Chiu K.W., Lee F.A., Kong F.S., Chan A.C. (2021). Combined Stereotactic Body Radiotherapy and Immunotherapy Versus Transarterial Chemoembolization in Locally Advanced Hepatocellular Carcinoma: A Propensity Score Matching Analysis. Front. Oncol..

[113] Li J.X., Su T.S., Gong W.F., Zhong J.H., Yan L.Y., Zhang J., Li L.Q., He M.L., Zhang R.J., Du Y.Q. (2022). Combining stereotactic body radiotherapy with camrelizumab for unresectable hepatocellular carcinoma: A single-arm trial. Hepatol. Int..

[114] Su C.W., Hou M.M., Huang P.W., Chou Y.C., Huang B.S., Tseng J.H., Hsu C.W., Chang T.C., Lin S.M., Lin C.C. (2022). Proton beam radiotherapy combined with anti-PD1/PDL1 immune checkpoint inhibitors for advanced hepatocellular carcinoma. Am. J. Cancer Res..

[115] Su K., Guo L., Ma W., Wang J., Xie Y., Rao M., Zhang J., Li X., Wen L., Li B. (2022). PD-1 inhibitors plus anti-angiogenic therapy with or without intensity-modulated radiotherapy for advanced hepatocellular carcinoma: A propensity score matching study. Front. Immunol..

[116] Huang Y., Zhang Z., Liao W., Hu K., Wang Z. (2021). Combination of Sorafenib, Camrelizumab, Transcatheter Arterial Chemoembolization, and Stereotactic Body Radiation Therapy as a Novel Downstaging Strategy in Advanced Hepatocellular Carcinoma With Portal Vein Tumor Thrombus: A Case Series Study. Front. Oncol..

[117] Chen Y., Hong H., Fang W., Zhang X., Luo H., Chen Z., Yu J., Fan W., Chi X., Peng Y. (2023). Toripalimab in combination with Anlotinib for unresectable hepatocellular carcinoma after SBRT: A prospective, single-arm, single-center clinical study. Front. Oncol..

